# GZ17-6.02 and Pemetrexed Interact to Kill Osimertinib-Resistant NSCLC Cells That Express Mutant ERBB1 Proteins

**DOI:** 10.3389/fonc.2021.711043

**Published:** 2021-08-19

**Authors:** Laurence Booth, Cameron West, Robert P. Moore, Daniel Von Hoff, Paul Dent

**Affiliations:** ^1^Department of Biochemistry and Molecular Biology, Virginia Commonwealth University, Richmond, VA, United States; ^2^Genzada Pharmaceuticals, Sterling, KS, United States; ^3^Translational Genomics Research Institute (TGEN), Phoenix, AZ, United States

**Keywords:** GZ17-6.02, pemetrexed, osimertinib, NSCLC, resistance, autophagy, EGFR, ER stress

## Abstract

We determined the molecular mechanisms by which the novel therapeutic GZ17-6.02 killed non-small cell lung cancer (NSCLC) cells. Erlotinib, afatinib, and osimertinib interacted with GZ17-6.02 to kill NSCLC cells expressing mutant EGFR proteins. GZ17-6.02 did not interact with any EGFR inhibitor to kill osimertinib-resistant cells. GZ17-6.02 interacted with the thymidylate synthase inhibitor pemetrexed to kill NSCLC cells expressing mutant ERBB1 proteins or mutant RAS proteins or cells that were resistant to EGFR inhibitors. The drugs interacted to activate ATM, the AMPK, and ULK1 and inactivate mTORC1, mTORC2, ERK1/2, AKT, eIF2α; and c-SRC. Knockdown of ATM or AMPKα_1_ prevented ULK1 activation. The drugs interacted to cause autophagosome formation followed by flux, which was significantly reduced by knockdown of ATM, AMPKα_1_, and eIF2α, or by expression of an activated mTOR protein. Knockdown of Beclin1, ATG5, or [BAX + BAK] partially though significantly reduced drug combination lethality as did expression of activated mTOR/AKT/MEK1 or over-expression of BCL-XL. Expression of dominant negative caspase 9 weakly reduced killing. The drug combination reduced the expression of HDAC2 and HDAC3, which correlated with lower PD-L1, IDO1, and ODC levels and increased MHCA expression. Collectively, our data support consideration of combining GZ17-6.02 and pemetrexed in osimertinib-resistant NSCLC.

## Introduction

The drug GZ17-6.02 is undergoing phase I evaluation in solid tumor patients (NCT03775525). GZ17-6.02 has three components that are natural chemicals: curcumin (10%), isovanillin (77%), and harmine (13%) (1–4). The most widely studied compound is curcumin, i.e., turmeric, the spice most associated with Indian cuisine, which is composed of ~95% of curcumin and curcuminoid derivatives. The safe maximal plasma concentration of commercially available lecithin liposomal curcumin, e.g., Meriva^®^, for an 800-mg ingestion is approximately 2 µM. Our prior *in vitro* studies have used GZ17-6.02 with the basal concentration of curcumin set at 2.0 μM (1–3). The plants *Arum palaestinum* and *Peganum harmala* have been used for centuries in the Levant and Orient for the treatment of many ailments, including cancer (5–9). The most bio-active chemical isolated from these plants is harmine. Studies have shown that while harmine has anti-proliferative effects in tumor cells, the compound appears to lack any anti-proliferative biologic effects in non-transformed cells. We have previously shown that GZ17-6.02 interacted with 5-fluorouracil (5FU) to kill GI tumor cells, with doxorubicin to kill sarcoma cells and with [trametinib + dabrafenib] to kill cutaneous melanoma cells expressing B-RAF V600E (1, 2). Our new studies were performed to determine whether GZ17-6.02 could kill non-small cell lung cancer (NSCLC) cells expressing mutant activated forms of the EGF receptor (ERBB1).

The treatment of NSCLC over the past 20 years has been revolutionized, first by the development of the pemetrexed carboplatin drug combination and then subsequently by checkpoint inhibitory immunotherapy (10–14). For NSCLC tumors expressing mutant RAS proteins or without a clear oncogenic driver, the combination of pemetrexed, carboplatin, and an anti-PD1 antibody, e.g., pembrolizumab, is a standard of care therapeutic approach. A subset of NSCLC patients present with tumors whose biology is driven by expression of mutated active forms of ERBB1. Some of the mutant ERBB1 proteins are point mutation mutants and others are deletion mutants (10, 11). Multiple ERBB1 inhibitors are approved to treat this form of the disease including erlotinib, afatinib, and recently osimertinib. Osimertinib is a relatively specific inhibitor of mutant active forms of ERBB1 and is, at present, the standard of care therapeutic. As with all targeted drugs in cancer, eventually NSCLC cells become osimertinib resistant, with diverse mechanisms, including gain of additional ERBB1 mutations or activation of other receptor tyrosine kinases such as c-MET and FGFRs (15–18). Overcoming osimertinib resistance remains an important area for the developmental cancer therapeutics field in NSCLC.

The studies in the present manuscript initially determined whether GZ17-6.02 interacted with ERBB1 inhibitors to kill NSCLC cells expressing mutant ERBB1 proteins. Subsequently, we determined how GZ17-6.02 killed osimertinib-resistant NSCLC cells and interacted with the standard of care agent pemetrexed to further enhance killing.

## Materials and Methods

### Materials

All human NSCLC lines were obtained from the ATCC (Bethesda, MD). Lewis Lung Carcinoma cells were obtained from the NCI repository (Bethesda, MD). Pemetrexed, erlotinib, afatinib, and osimertinib were purchased from Selleckchem (Houston, TX). All materials were obtained as described in the references (19–24). Trypsin-EDTA, DMEM, RPMI, and penicillin-streptomycin were purchased from GIBCOBRL (GIBCOBRL Life Technologies, Grand Island, NY). The kit to assess GSH levels and the GSH : GSSG ratio was purchased from Promega (GSH/GSSG-Glo Assay; Madison WI) and assays were performed as per the kit instructions. Other reagents and performance of experimental procedures were as described (19–24). Antibodies used: AIF (5318), BAX (5023), BAK (12105), BAD (9239), BIM (2933), BAK1 (12105), Beclin1 (3495), cathepsin B (31718), CD95 (8023), FADD (2782), eIF2α (5324), P-eIF2α S51 (3398), ULK-1 (8054), P-ULK-1 S757 (14202), P-AMPK S51 (2535), AMPKα (2532), P-ATM S1981 (13050), ATM (2873), ATG5 (12994), mTOR (2983), P-mTOR S2448 (5536), P-mTOR S2481 (2974), ATG13 (13468), MCL-1 (94296), BCL-XL (2764), P-AKT T308 (13038), P-ERK1/2 (5726), P-STAT3 Y705 (9145), P-p65 S536 (3033), p62 (23214), and LAMP2 (49067) all from Cell Signaling Technology (Danvers, MA); P-ULK-1 S317 (3803a) was from Abgent; P-ATG13 S318 (19127) was from Novus Biologicals. Anti-PD-L1, PD-L2, and MHCA antibodies were from ABCAM (Cambridge, UK). The ODC antibody was purchased from Santa Cruz Biotechnology (Dallas, TX). Specific multiple independent siRNAs to knock down the expression of CD95, FADD, Beclin1, ATG5, and eIF2α, and scramble control, were purchased from Qiagen (Hilden Germany). Control studies were presented showing on-target specificity of our siRNAs, primary antibodies, and our phospho-specific antibodies to detect both total protein levels and phosphorylated levels of proteins (1–3, 19–24) (**Supplementary Figure S1**).

### Methods

All bench-side methods used in this manuscript have been performed and described in the peer-reviewed references (1–3, 19–24). All cell lines were cultured at 37°C (5% (v/v CO_2_) *in vitro* using RPMI supplemented with dialyzed 5% (v/v) fetal calf serum and 1% (v/v) Non-essential amino acids. Drugs are dissolved in DMSO to make 10 mM stock solutions. The stock solution is diluted to the desired concentration in the media that the cells being investigated grow in. We ensure that the concentration of DMSO is never more than 0.1% (v/v) in the final dilution that is added to cells, to avoid solvent effects. Cells were not cultured in reduced serum media during any study in this manuscript.

### Generation of Erlotinib, Afatinib, and Osimertinib-Resistant Cells

Cells were incubated *in vitro* with increasing concentrations of vehicle control or erlotinib or osimertinib until after ~6 weeks the HCC827 and H1975 and H1650 cells grew with similar kinetics to sensitive cells in either erlotinib (1 μM) or osimertinib (1 μM), respectively. Afatinib-resistant cells were created *in vivo* by repeated high dosing until tumors disappeared and then regrew, as previously described (25).

### Assessments of Protein Expression and Protein Phosphorylation

Multi-channel fluorescence HCS microscopes perform true in-cell Western blotting. Three independent cultures derived from three thawed vials of cells of a tumor were sub-cultured into individual 96-well plates. Twenty-four hours after plating, the cells are transfected with a control plasmid or a control siRNA, or with an empty vector plasmid or with plasmids to express various proteins. After another 24 h, the cells are ready for drug exposure(s). At various time points after the initiation of drug exposure, cells are fixed in place using paraformaldehyde and using Triton X-100 for permeabilization. Standard immunofluorescent blocking procedures are employed, followed by incubation of different wells with a variety of validated primary antibodies and subsequently validated fluorescent-tagged secondary antibodies are added to each well. The microscope determines the background fluorescence in the well and in parallel randomly determines the mean fluorescent intensity of 100 cells per well. Of note for scientific rigor is that the operator does not personally manipulate the microscope to examine specific cells; the entire fluorescent accrual method is independent of the operator.

For co-localization studies, three to four images of cells stained in the red and green fluorescence channels are taken for each treatment/transfection/condition. Images are approximately 4 MB sized files. Images are merged in Adobe Photoshop CS5, and the image intensity and contrast is then post-hoc altered in an identical fashion inclusive for each group of images/treatments/conditions, so that the image with the weakest intensity is still visible to the naked eye for publication purposes but also that the image with the highest intensity is still within the dynamic range, i.e., not over-saturated.

### Detection of Cell Death by Trypan Blue Assay

Cells were treated with vehicle control or with drugs alone or in combination for 24 h. At the indicated time points, cells were harvested by trypsinization and centrifugation. Cell pellets were resuspended in PBS and mixed with trypan blue agent. Viability was determined microscopically using a hemocytometer. Five hundred cells from randomly chosen fields were counted and the number of dead cells was counted and expressed as a percentage of the total number of cells counted.

### Transfection of Cells With siRNA or With Plasmids

#### For Plasmids

Cells were plated and, 24 h after plating, transfected. Plasmids to express FLIP-s, BCL-XL, dominant negative caspase 9, activated AKT, activated mTOR, and activated MEK1 EE were used throughout the study (Addgene, Waltham, MA). Empty vector plasmid (CMV) was used as a control. Plasmids expressing a specific mRNA or appropriate empty vector control plasmid (CMV) DNA was diluted in 50 μl of serum-free and antibiotic-free medium (one portion for each sample). Concurrently, 2 μl of Lipofectamine 2000 (Invitrogen) was diluted into 50 μl of serum-free and antibiotic-free medium (one portion for each sample). Diluted DNA was added to the diluted Lipofectamine 2000 for each sample and incubated at room temperature for 30 min. This mixture was added to each well/dish of cells containing 100 μl of serum-free and antibiotic-free medium for a total volume of 300 μl, and the cells were incubated for 4 h at 37°C. An equal volume of 2× serum-containing medium was then added to each well. Cells were incubated for 24 h and then treated with drugs.

#### Transfection for siRNA

Cells from a fresh culture growing in log phase as described above and 24 h after plating were transfected. Prior to transfection, the medium was aspirated, and serum-free medium was added to each plate. For transfection, 10 nM of the annealed siRNA or the negative control (a “scrambled” sequence with no significant homology to any known gene sequences from mouse, rat or human cell lines) were used. Ten nanomolar siRNA (scrambled or experimental) was diluted in serum-free media. Four milliliters of Hiperfect (Qiagen) was added to this mixture and the solution was mixed by pipetting up and down several times. This solution was incubated at room temperature for 10 min and then added dropwise to each dish. The medium in each dish was swirled gently to mix and then incubated at 37°C for 2 h. Serum-containing medium was added to each plate, and cells were incubated at 37°C for 24 h before and then treated with drugs (0–24 h).

#### Assessments of Autophagosome and Autolysosome Levels

Cells were transfected with a plasmid to express LC3-GFP-RFP (Addgene, Watertown MA). Twenty-four hours after transfection, cells are treated with vehicle control or the drugs alone or in combination. Cells were imaged and recorded at 60× magnification 4 h and 8 h after drug exposure and the mean number of GFP+ and RFP+ punctae per cell was determined from >50 randomly selected cells per condition.

#### Data Analysis

Comparison of the effects of various treatments was done using one-way ANOVA for normalcy followed by a two tailed Student’s *t*-test with multiple comparisons. Differences with a *p*-value of <0.05 were considered statistically significant. Experiments are the means of multiple individual data points per experiment from three independent experiments (± SD).

## Results

### GZ17-6.02 Interacts With ERBB1 Inhibitors to Kill NSCLC Cells

**Figure 1:**
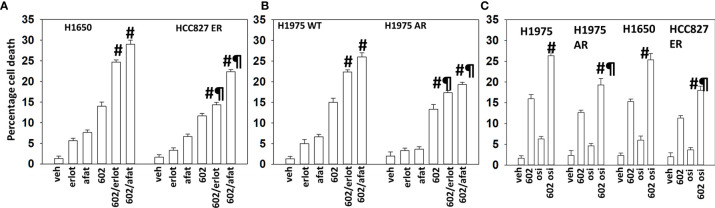
GZ17-6.02 interacts with ERBB1 inhibitors to kill NSCLC cells expressing mutant active forms of ERBB1. **(A, B)** H1650, wild-type sensitive, and afatinib-resistant (AR) H1975 and erlotinib-resistant (ER) HCC827 cells were treated with vehicle, erlotinib (500 nM), afatinib (500 nM), GZ17-6.02 (2 μM curcumin final), or the drugs in combination for 24 h. Cell viability was determined by trypan blue exclusion (*n* = 3 ± SD). ^#^ *p* < 0.05 greater than GZ17-6.02 alone; ^¶^ *p* < 0.05 less than the corresponding values in drug-sensitive cells. **(C)** H1650, wild-type sensitive, and afatinib-resistant (AR) H1975 and erlotinib-resistant (ER) HCC827 cells were treated with vehicle, osimertinib (100 nM), GZ17-6.02 (2 μM curcumin final), or the drugs in combination for 24 h. Cell viability was determined by trypan blue exclusion (*n* = 3 ± SD). ^#^ *p* < 0.05 greater than GZ17-6.02 alone; ^¶^ *p* < 0.05 less than the corresponding values in drug-sensitive cells.

GZ17-6.02 interacted with erlotinib, afatinib, and osimertinib to kill H1975 and H1650 cells that express mutant activated ERBB1 proteins (**Figures 1A–C**). In erlotinib HCC827 cells, the abilities of erlotinib and afatinib to enhance GZ17-6.02 lethality were significantly reduced as was also observed in afatinib-resistant H1975 cells (**Figures 1A, B**). The ability of osimertinib to enhance the efficacy of GZ17-6.02 was also reduced in afatinib- and erlotinib-resistant cells (**Figure 1C**).

### GZ17-6.02 Kills Osimertinib-Resistant NSCLC Cells

**Figure 2:**
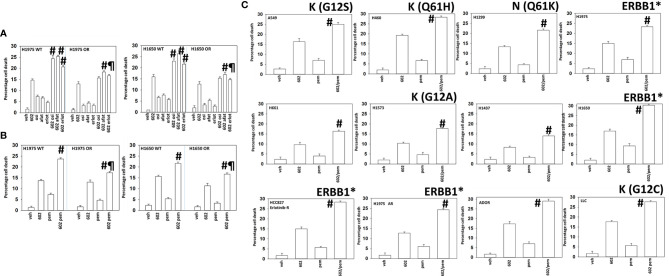
GZ17-6.02 interacts with pemetrexed to kill NSCLC cells. **(A)** H1975 and H1650 cells [wild-type sensitive and osimertinib resistant (OR)] were treated with vehicle, erlotinib (500 nM), afatinib (500 nM), osimertinib (100 nM), GZ17-6.02 (2 μM curcumin final), or the drugs in combination for 24 h. Cell viability was determined by trypan blue exclusion (*n* = 3 ± SD). ^#^ *p* < 0.05 greater than GZ17-6.02 alone; ^¶^ *p* < 0.05 less than the corresponding values in drug-sensitive cells. **(B)** H1975 and H1650 cells [wild-type sensitive and osimertinib resistant (OR)] were treated with vehicle, pemetrexed (500 nM), GZ17-6.02 (2 μM curcumin final), or the drugs in combination for 24 h. Cell viability was determined by trypan blue exclusion (*n* = 3 ± SD). ^#^ *p* < 0.05 greater than GZ17-6.02 alone; ^¶^ *p* < 0.05 less than the corresponding values in drug-sensitive cells. **(C)** NSCLC cells were treated with vehicle, pemetrexed (500 nM), GZ17-6.02 (2 μM curcumin final), or the drugs in combination for 24 h. Cell viability was determined by trypan blue exclusion (*n* = 3 ± SD). ^#^ *p* < 0.05 greater than GZ17-6.02 alone. The mutational status of K-/N-RAS or of ERBB1 is noted in each graph. * = mutated active

We generated osimertinib-resistant H1975 and H1650 cells, as described in the *Methods*. In osimertinib-resistant cells, the abilities of erlotinib and osimertinib to enhance GZ17-6.02 killing were abolished, with only afatinib capable of modestly enhancing tumor cell killing (**Figure 2A**). We next determined whether GZ17-6.02 could interact with the NSCLC therapeutic pemetrexed to kill wild-type and osimertinib-resistant cells. As noted in **Figure 2A**, osimertinib resistance weakly reduced the efficacy of GZ17-6.02 as a single agent, and it interacted to kill both wild-type and osimertinib-resistant cells, albeit with a lesser efficacy in the resistant cells (**Figure 2B**). We then determined whether GZ17-6.02 interacted with pemetrexed to kill other NSCLC cell lines, regardless of mutant RAS or ERBB1 expression; GZ17-6.02 and pemetrexed interacted to kill (**Figure 2C**).

### GZ17-6.02 and Osimertinib Interact to Inactivate mTOR and eIF2a

We then determined the alterations in cellular signaling and protein expression in NSCLC cells treated with GZ17-6.02 and either osimertinib or pemetrexed. GZ17-6.02 interacted with osimertinib in wild-type H1975 cells to activate ATM, the AMPK, ULK1, ATG13, and PERK (**Supplementary Tables S1** and **S2**). The drugs interacted to cause inactivation of mTORC1, mTORC2, eIF2α, MEK1/2, ERK1/2, AKT, JAK2, STAT3, STAT5, ERBB1, PDGFRβ, c-MET, p70 S6K, c-SRC, NFκB, JNK1/2, YAP, and TAZ. The drug combination increased protein MHCA expression and reduced the levels of PD-L1, IDO1, HDAC1, HDAC2, HDAC3, HDAC4, HDAC6, and HDAC7. Similar findings were made in H1650 cells. In afatinib-resistant H1975 cells, the drug combination caused significantly more ERK1/2 inactivation and did not inactivate p70 S6K or STAT5 and caused a compensatory increase in c-KIT survival signaling (that was not observed in osimertinib-resistant cells).

### GZ17-6.02 and Osimertinib Interact to Cause Autophagosome Formation Followed by Autophagic Flux

Based on our prior studies with GZ17-6.02, we predicted that the inactivation of mTOR, the activation of ULK1, and increased ATG13 S318 phosphorylation would cause autophagosome formation (1–3). GZ17-6.02 interacted with osimertinib in an additive fashion to increase autophagosome formation and subsequently autophagosome formation (**Supplementary Figure S2A**, upper graph). In afatinib-resistant H1975 cells, GZ17-6.02 enhanced autophagosome formation to a lesser extent than in wild-type sensitive cells and did not further interact with osimertinib (lower graph). Increasing numbers of autolysosomes were also observed 8 h after treatment, but again, this value was lower than that observed in the sensitive cells. Knockdown of Beclin1 or ATG5 prevented the initial increase in autophagosome levels and the subsequent increase in autolysosome levels (not shown).

### Autophagosome Formation and Autophagic Flux Play Key Roles in Causing Tumor Cell Death

We next determined the relative role of altered cellular signaling processes in autophagosome formation and autophagic flux. Knockdown of ATM, AMPKα, eIF2α; or expression of activated mTOR or activated STAT3 significantly suppressed autophagosome formation and autophagic flux (**Supplementary Figures S2B, C**). Knockdown of [BAX + BAK], Beclin1, ATG5, or FADD significantly reduced cell killing by [GZ17-6.02 + osimertinib] (**Supplementary Figure S3**). The total levels of GSH and the GSH : GSSG ratio were not significantly altered by GZ17-6.02 over 12 h (**Supplementary Figure S4**). Modest significant reductions in the levels of GSH and alterations in the ratio were observed after 24–48 h; however, there was no clear dose dependency comparing the two GZ17-6.02 concentrations. These data imply that autophagy, death receptor signaling, and mitochondrial dysfunction play key roles in the cell killing caused by the drug combination, with altered redox potential unlikely to play any role. Of note was that expression of dominant negative caspase 9 relatively weakly prevented cell death compared to other interventions arguing that non-apoptotic processes downstream of the mitochondrion played key roles.

### Regardless of ERBB1 Inhibitor Resistance, GZ17-6.02 and Pemetrexed Regulate Cell Signaling in a Near-Identical Fashion

Based on our viability data with GZ17-6.02 and pemetrexed in ERBB1 inhibitor-resistant NSCLC cells, we compared and contrasted the ability of the drug combination to alter signaling and protein expression in H1975 cells; wild-type sensitive; afatinib-resistant; and osimertinib-resistant. Regardless of drug resistance, the drug combination activated ATM, AMPK, ULK1, ATG13, and PERK. The combination inactivated ERBB1, ERBB2, mTORC1, mTORC2, eIF2α, AKT, ERK1/2, JAK2, STAT3, STAT5, p70 S6K, NFκB, c-SRC, c-MET, and c-KIT. The combination increased the expression of Beclin1, ATG5, and FAS-L and reduced the expression of BCL-XL and MCL1 (**Supplementary Tables S3** and **S4**). Regardless of osimertinib resistance, the drug combination reduced the protein levels of HDAC2, HDAC3, and HDAC6 (**Supplementary Table S5**). In prior work, we have linked reduced expression of HDAC2 and HDAC3 to increased expression of the immunotherapy biomarker MHCA and reduced levels of PD-L1. In multiple NSCLC lines, the drug combination significantly reduced expression of PD-L1, ODC, and IDO1 and elevated MHCA levels (**Supplementary Table S6**).

### GZ17-6.02 and Pemetrexed Interact to Increase Autophagy

**Figure 3:**
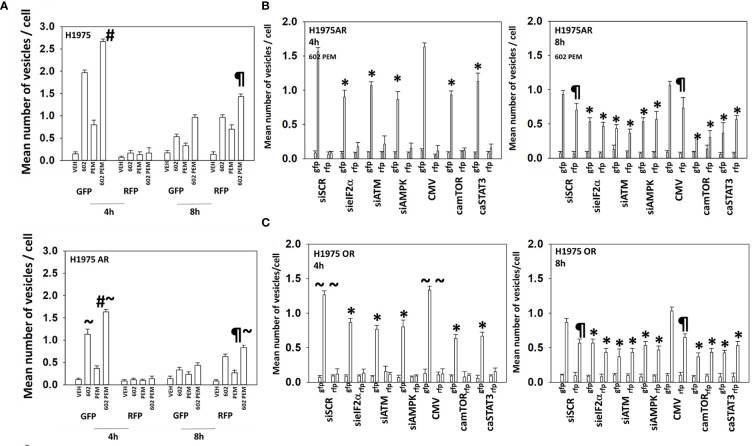
Resistance to ERBB1 inhibitors is associated with a reduced ability to form autophagosomes. **(A)** H1975 [wild-type sensitive and afatinib-resistant (AR)] were transfected to express LC3-GFP-RFP and subsequently treated with vehicle, pemetrexed (500 nM), GZ17-6.02 (2 μM curcumin final), or the drugs in combination for 4 h and 8 h. The number of intense staining GFP+ and RFP+ punctae was determined randomly in at least 50 cells, and the mean number of punctae per cell was determined (*n* = 3 ± SD). ^#^ *p* < 0.05 greater than GZ17-6.02 value; ^¶^ *p* < 0.05 greater than the corresponding values after 4 h; ~*p* < 0.05 less than the corresponding values in wild-type sensitive cells. **(B)** Afatinib-resistant H1975 cells were transfected with siRNA molecules to knock down protein levels or with plasmids to express activated forms of mTOR or STAT3 and then subsequently treated with vehicle or [pemetrexed (500 nM) + GZ17-6.02 (2 μM curcumin final)] in combination for 4 h and 8 h. The number of intense staining GFP+ and RFP+ punctae were determined randomly in at least 50 cells, and the mean number of punctae per cell was determined (*n* = 3 ± SD). ^¶^ *p* < 0.05 greater than the corresponding values after 4 h; *p < 0.05 less than the corresponding values in siSCR/CMV-transfected cells. **(C)** Osimertinib-resistant H1975 cells were transfected with siRNA molecules to knock down protein levels or with plasmids to express activated forms of mTOR or STAT3 and then subsequently treated with vehicle or [pemetrexed (500 nM) + GZ17-6.02 (2 μM curcumin final)] in combination for 4 h and 8 h. The number of intense staining GFP+ and RFP+ punctae was determined randomly in at least 50 cells and the mean number of punctae per cell determined (*n* = 3 ± SD). ^¶^ *p* < 0.05 greater than the corresponding values after 4 h; ~~*p* < 0.05 less than the corresponding values in afatinib-resistant H1975 cells; *p < 0.05 less than the corresponding values in siSCR/CMV-transfected cells.

**Figure 4:**
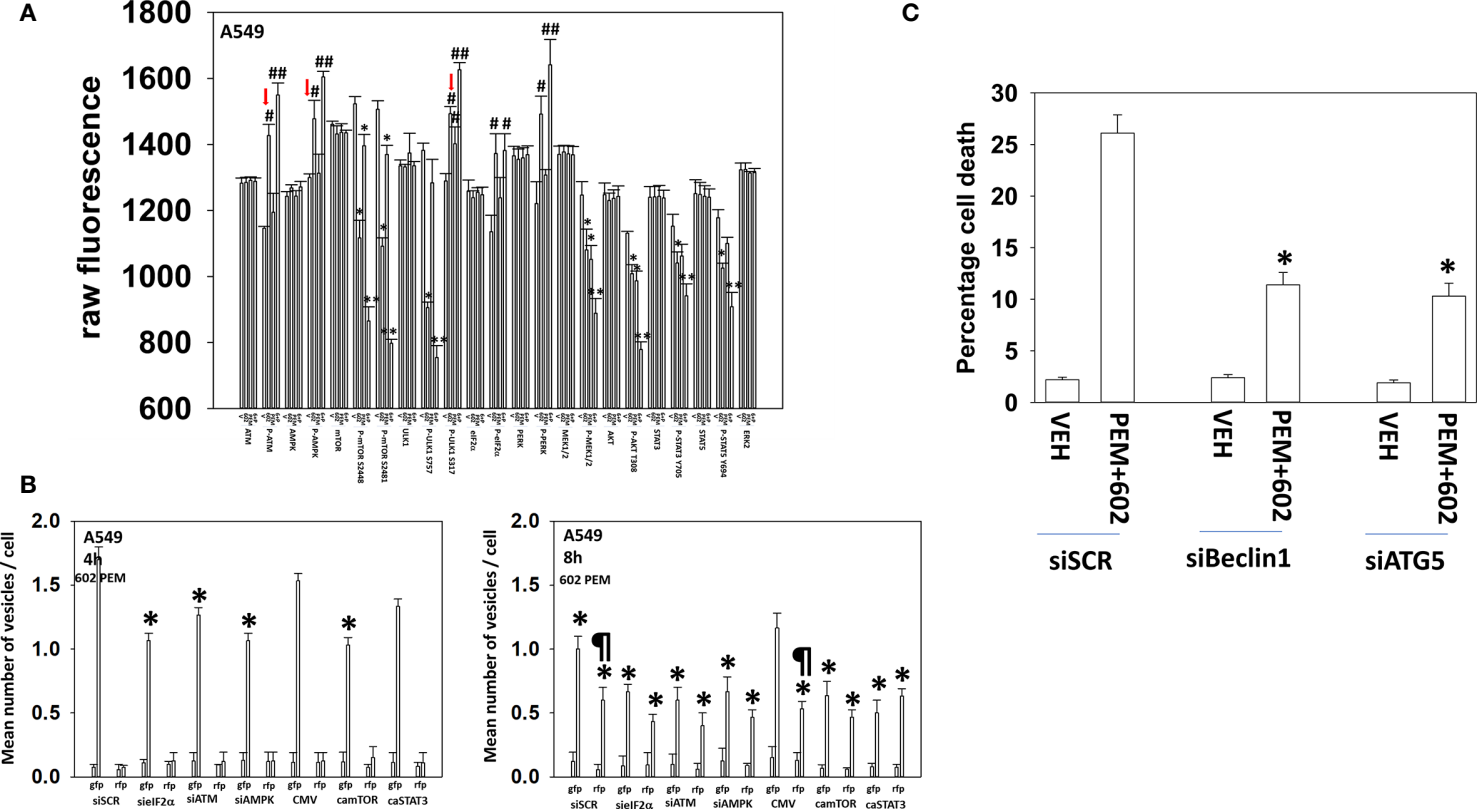
GZ17-6.02 and pemetrexed interact to alter cell signaling, increase autophagosome formation, and kill *via* toxic autophagy A549 NSCLC cells that express a mutant K-RAS protein. **(A)** A549 cells were treated with vehicle control, GZ17-6.02 (2 μM final curcumin), pemetrexed (500 nM), or the drugs combined for 6 h. Cells were fixed in place and immunostaining was performed to determine protein expression and phosphorylation (*n* = 3 ± SD) *p < 0.05 less than vehicle; **p < 0.05 less than GZ17-6.02 alone; ^#^ *p* < 0.05 greater than vehicle control; ^##^ *p* < 0.05 greater than GZ17-6.02 alone. **(B)** A549 cells were transfected with siRNA molecules to knock down protein levels or with plasmids to express activated forms of mTOR or STAT3 and then subsequently treated with vehicle or [pemetrexed (500 nM) + GZ17-6.02 (2 μM curcumin final)] in combination for 4 h and 8 h. The number of intense staining GFP+ and RFP+ punctae were determined randomly in at least 50 cells, and the mean number of punctae per cell was determined (*n* = 3 ± SD). ^¶^ *p* < 0.05 greater than the corresponding values after 4 h; *p < 0.05 less than the corresponding values in siSCR/CMV-transfected cells. **(C)** A549 cells were transfected to knock down Beclin1 or ATG5 expression. Subsequently cells were treated with vehicle, pemetrexed (500 nM), GZ17-6.02 (2 μM curcumin final), or the drugs in combination for 24 h. Cell viability was determined by trypan blue exclusion (*n* = 3 ± SD). *p < 0.05 less than the corresponding values in siSCR cells.

GZ17-6.02 interacted with pemetrexed in an additive fashion to increase autophagosome formation and to cause autophagic flux (**Figure 3A**). The drug combination caused significantly less autophagosome formation and autophagic flux in the afatinib-resistant cells. The ability of afatinib-resistant cells to form autophagosomes after drug exposure was significantly reduced by knockdown of eIF2α, ATM, or AMPKα or by expression of activated mTOR or activated STAT3 (**Figure 3B**). The ability of [GZ17-6.02 + pemetrexed] to cause autophagosome formation in the osimertinib-resistant cells was significantly lower than that found in wild-type sensitive or afatinib-resistant cells (**Figure 3C**). Autophagosome formation in the osimertinib-resistant cells was also significantly reduced by knockdown of eIF2α, ATM, or AMPKα or by expression of activated mTOR or activated STAT3. In contrast to our autophagosome data, the drug-induced levels of autolysosomes in the afatinib-resistant and osimertinib-resistant cells were not significantly different. Similar alterations in cell signaling, autophagy, and viability data were obtained treating A549 NSCLC cells with the drug combination that expresses a mutant K-RAS protein and erlotinib-resistant HCC827 cells (**Figure 4**; **Supplementary Figure S5**). In contrast to the other lines tested, the HCC827 line exhibited a strong dependence on altered signaling by ATM and mTOR to stimulate autophagosome formation.

### GZ17-6.02 and Pemetrexed Use Autophagy to Kill NSCLC Cells

**Figure 5:**
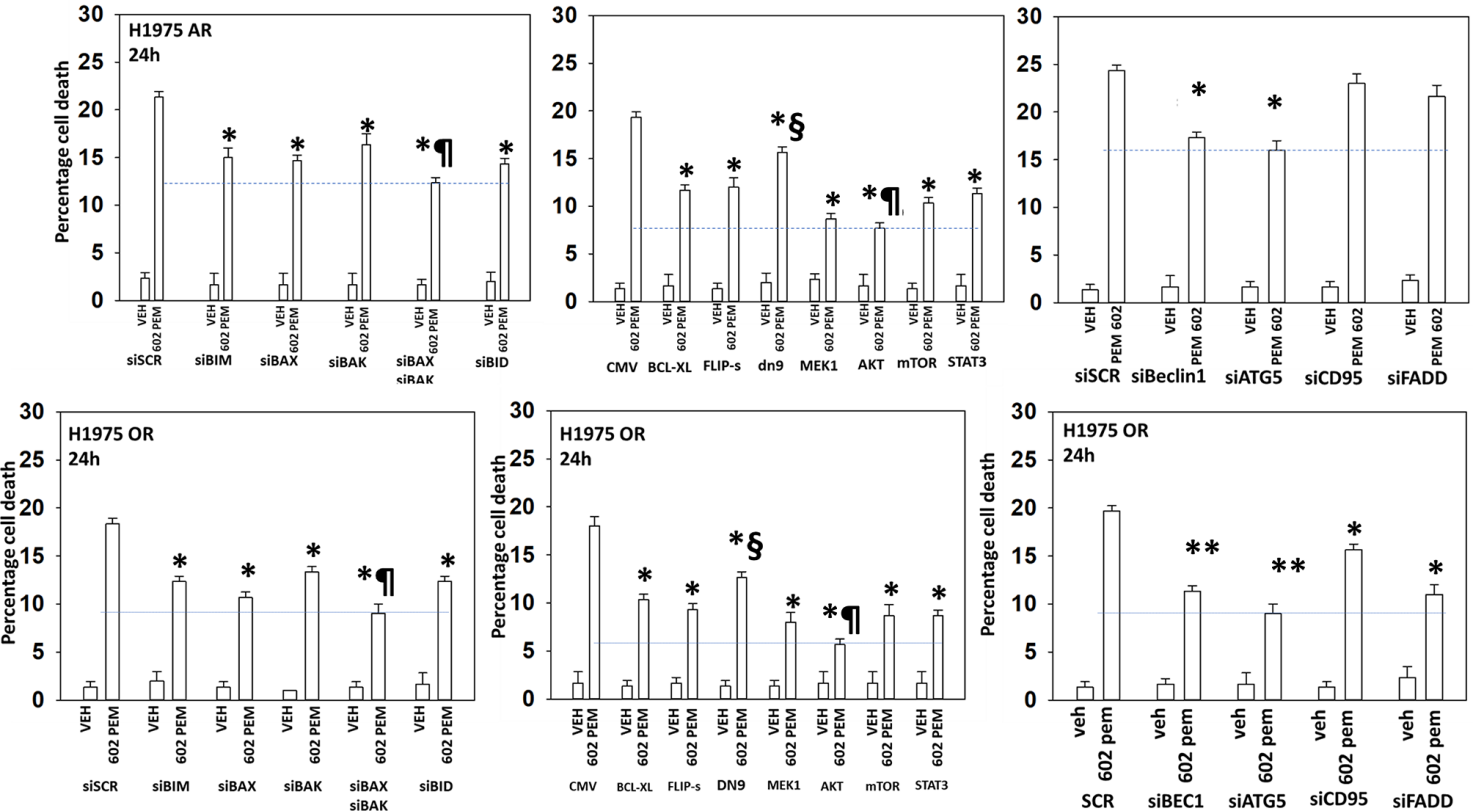
The killing of osimertinib-resistant NSCLC cells requires [BAX + BAK] and autophagosome formation and is significantly reduced by expression of activated AKT, activated mTOR or activated MEK1. Afatinib-resistant and osimertinib-resistant H1975 cells were transfected with siRNA molecules to knock down protein expression or with plasmids to express regulatory proteins. Subsequently, cells were treated with vehicle or [pemetrexed (500 nM) + GZ17-6.02 (2 μM curcumin final)] in combination for 24 h. Cell viability was determined by trypan blue exclusion (*n* = 3 ± SD). *p < 0.05 less than corresponding siSCR/CMV value; **p < 0.05 less than the corresponding values in afatinib-resistant cells; ^¶^ *p* < 0.05 less than the corresponding values in all other conditions; ^§^ *p* < 0.05 greater than the corresponding values in all other manipulated conditions.

The ability of [GZ17-6.02 + pemetrexed] to kill osimertinib-resistant cells trended lower than the ability of the drug combination to kill afatinib-resistant cells (**Figures 4** and **5**). Combined knockdown of BAX and BAK significantly reduced killing in both the afatinib-resistant and the osimertinib-resistant cells by ~50% with knockdown of BID reducing death by ~35%. In both resistant cell types, activated AKT and, to a lesser extent, activated MEK1, activated STAT3, or activated mTOR significantly reduced killing. Knockdown of Beclin1 or ATG5 was significantly more protective in osimertinib-resistant cells compared to afatinib-resistant cells. Knockdown of Beclin1 or ATG5 prevented the initial increase in autophagosome levels and the subsequent increase in autolysosome levels (not shown). Death receptor signaling also trended to be more important in the killing processes in osimertinib-resistant cells than in afatinib-resistant cells. Expression of dominant negative caspase 9 was less protective than over-expression of FLIP-s or BCL-XL in both resistant lines arguing that cell execution downstream of the mitochondrion was largely non-apoptotic.

## Discussion

The development of drug resistance in NSCLC tumors expressing mutant active forms of ERBB1 is a major problem in prolonging patient quality of life and survival. The present studies were designed to define the biology of GZ17-6.02 in NSCLC cells expressing mutant active ERBB1 proteins and to define whether it could overcome resistance to afatinib or osimertinib. GZ17-6.02 interacted with erlotinib, afatinib, or osimertinib to kill NSCLC cells expressing mutant ERBB1. However, in cells made resistant to either afatinib or osimertinib, GZ17-6.02 could not subvert the resistant phenotype. Based on those findings, we then determined whether GZ17-6.02 interacted with the NSCLC therapeutic pemetrexed to kill. Resistance to ERBB1 inhibitors only modestly reduced the efficacy of GZ17-6.02 and caused only a ~20% reduction in the lethal interaction between GZ17-6.02 and pemetrexed.

When we examined drug-induced changes in cell signaling in the sensitive and ERBB1 inhibitor resistant cells, combining GZ17-6.02 with either osimertinib or pemetrexed, their responses exhibited subtle rather than profound differences. For example, from over 20 parameters measured, the major observation for afatinib-resistant cells treated with [GZ17-6.02 + osimertinib] was that the drug combination caused significantly more ERK1/2 inactivation in sensitive cells and did not inactivate p70 S6K or STAT5 and that it caused a compensatory increase in c-KIT survival signaling in resistant cells. The complex milieux of signaling trends collectively resulted in the outcomes of afatinib-resistant cells being less capable to form autophagosomes and to die.

Because our initial hypotheses were incorrect regarding the hope that GZ17-6.02 would abolish ERBB1 inhibitor resistance, we then performed studies to define the interactions of GZ17-6.02 with the standard of care therapeutic pemetrexed in the NSCLC cells. We specifically chose pemetrexed rather than carboplatin because *via* DNA damage signaling, pemetrexed causes ATM activation, and by increasing the intracellular concentration of ZMP, and analogue of AMP, it causes allosteric activation of the AMPK (26–28). In wild-type sensitive cells compared to osimertinib-resistant cells, [GZ17-6.02 + pemetrexed] signaling trended to cause greater inactivation of ERBB1 and ERBB2, whereas in the osimertinib-resistant cells, greater ERBB4 and c-MET inactivation was observed.

Regardless of ERBB1 inhibitor resistance, [GZ17-6.02 + pemetrexed] inactivated AKT, mTORC1, and mTORC2 to a similar extent. The amount of drug-induced ATG13 S318 phosphorylation induced was also identical regardless of drug resistance, as were the increased levels of Beclin1 and ATG5. Nevertheless, afatinib-resistant H1975 cells were significantly less efficient at forming autophagosomes than wild-type sensitive cells, a ~55% reduction, and osimertinib-resistant cells exhibited a further significant reduction in autophagosome formation compared to the afatinib-resistant cells. Both afatinib- and osimertinib-resistant cells exhibited similar levels of subsequent autolysosome formation, which was ~30% of that observed in the sensitive cells. Furthermore, knockdown of Beclin1 or ATG5 prevented the initial increase in autophagosome levels and the subsequent increase in autolysosome levels. These data argue that the “defect” in the drug-resistant cells is specifically related to autophagosome formation rather than the abilities of cells to promote autophagic flux and subsequent autolysosome formation. One potential mechanism by which autophagosome formation could be disrupted is *via* the sequestration of Beclin1 by protective BH3 domain proteins such as BCL-XL and MCL1. However, data from **Supplementary Table S4** demonstrated that the drug-resistant cells under basal conditions only expressed 10%–20% greater levels of BCL-XL than were found in the sensitive cells.

Over-expression of BCL-XL or knockdown of [BAX + BAK] significantly reduced tumor cell killing, implying that mitochondrial dysfunction played an important role in the killing process. In general agreement with prior studies using GZ17-6.02, the role of reactive oxygen species in the killing process appeared to be modest, as judged by the unaltered GSH : GSSG ratio. Mitochondria promote cell death downstream either through activation of caspase 9–caspase 3 signaling or directly *via* apoptosis-inducing factor (AIF). Expression of dominant negative caspase 9 relatively weakly prevented cell death compared to any of the other cyto-protective interventions arguing that non-apoptotic processes downstream of the mitochondrion played key roles.

In conclusion, *in vitro* and *in vivo*, GZ17-6.02 and pemetrexed interact to suppress the growth of osimertinib-resistant NSCLC cells and to prolong animal survival. Additional *in vitro* screening studies, beyond examination of Beclin1 and ATG5, will be required to understand why autophagosome formation is lower in osimertinib-resistant cells.

## Data Availability Statement

The original contributions presented in the study are included in the article/**Supplementary Material**. Further inquiries can be directed to the corresponding author.

## Author Contributions

LB performed the studies. PD wrote the manuscript. CW, PM, and DH provided guidance and advice and read the manuscript. All authors contributed to the article and approved the submitted version.

## Funding

Support for the present study was provided by Genzada Pharmaceuticals and by philanthropic funding from Massey Cancer Center and the Universal Inc. Chair in Signal Transduction Research.

## Conflict of Interest

PD has received funding from Genzada Pharmaceuticals Inc. PM and CW are paid officers of the company. PD and DH are key company scientific advisors/consultants.

The remaining author declares that the research was conducted in the absence of any commercial or financial relationships that could be construed as a potential conflict of interest

## Publisher’s Note

All claims expressed in this article are solely those of the authors and do not necessarily represent those of their affiliated organizations, or those of the publisher, the editors and the reviewers. Any product that may be evaluated in this article, or claim that may be made by its manufacturer, is not guaranteed or endorsed by the publisher.

## Abbreviations

ER, endoplasmic reticulum; AMPK, AMP-dependent protein kinase; mTOR, mammalian target of rapamycin; MAPK, mitogen-activated protein kinase; CMV, empty vector plasmid or virus; si, small interfering; SCR, scrambled; VEH, vehicle; PEM, pemetrexed; ERBB1/EGFR, epidermal growth factor receptor. ERK, extracellular regulated kinase; PI3K, phosphatidyl inositol 3 kinase; ca, constitutively active; dn, dominant negative; JAK, Janus Kinase; STAT, Signal Transducers and Activators of Transcription; VEH, vehicle; 602, GZ17-6.02; PD-L1, programed death ligand 1; HDAC, histone deacetylase; NSCLC, non-small cell lung cancer.
