# Supplementary material for: GZ17-6.02 and Pemetrexed Interact to Kill Osimertinib-Resistant NSCLC Cells That Express Mutant ERBB1 Proteins
Source: Front Oncol. 2021 Aug 19;11:711043. doi: 10.3389/fonc.2021.711043 (PMC8417372; doi:10.3389/fonc.2021.711043)

Vehicle 100%	1975 WT	602	OSI	6+O		1975 AR	602	OSI	6+O		1650 WT	602	OSI	6+O
P-ATM S1980		112	100	119#			112	101	116#			116#	98	118#
P-AMPKα T172		108	101	114#			108	102	115#			119#	98	120#
P-mTOR S2448		74*	68*	56*			69*	66*	60*			85*	96	83*
P-mTOR S2481		69*	68*	60*			68*	71*	60*			77*	87*	76*
P-ULK1 S757		68*	63*	58*			63*	65*	53**			77*	81*	71*
P-ULK1 S317		108	105	113#			106	99	118#			109	107	114#
P-PERK T980		118#	109	120#			117#	111	124#			114#	104	114#
P-eIF2α S51		115#	109	116#			116#	105	121#			117#	114	122#
P-MEK1/2		93	83*	76*			93	82*	76*			92	97	87*
P-AKT T308		91	81*	70*			94	86*	80*			95	90	85*
P-STAT3 Y705		93	92	85*			92	92	81*			96	95	94
P-STAT5 Y694		89	88	82*			93	95	92			95	98	93
Beclin1		110	113#	116#			106	109	115#			111	103	115#
ATG5		109	111	117#			107	105	113#			116#	106	117#
P-ATG13 S318		124#	126#	129#			118#	121#	126#			123#	115#	129#
GRP78		121#	119#	121#			115#	103#	116#			120#	111	122#
CHOP		114#	109	115#			110	102	113#			111	106	116#
PP1		116#	104	120#			119#	112	125#			119#	103	122#
NOXA		106	100	107			104	100	106			111	101	113#
PUMA		109	99	109			105	98	107			109	100	113#
FLIP-s		92	85*	84*			90	79*	75*			93	102	90
ERBB1		95	99	92			100	100	105			100	98	96
P-B1		94	73*	71*			93	76*	73*			71*	61*	58*
ERBB2		98	100	98			101	102	92			95	94	91
P-B2		91	101	91			93	101	91			74*	97	68*
ERBB3		97	101	100			101	101	101			100	100	100
P-B3		95	100	90			97	100	94			81*	96	76*
ERBB4		101	99	99			100	98	98			99	100	100
P-B4		94	102	93			100	97	94			75*	96	73*
p70 S6K T389		89	92	86*			102	99	91			87*	99	85*
Total ERK2		100	99	99			100	100	101			100	100	101

Supplemental Table 1. The impact of GZ17-6.02 and osimertinib on cell signaling in NSCLC cells (part 1). Cells were treated with vehicle control, GZ17-6.02 (2 μM final curcumin), osimertinib (100 nM) or the drugs combined for 6h. Cells were fixed in place and immunostaining performed to determine protein expression and phosphorylation (n = 3 +/-SD) * $p < 0.05$ less than vehicle; # $p < 0.05$ greater than vehicle.

Vehicle 100%	1975 WT	602	OSI	6+O		1975 AR	602	OSI	6+O		1650 WT	602	OSI	6+O
P-NFκB S536		89	100	86*			83*	102	82*			88	83*	80*
P-SRC Y416		83*	95	81*			86*	95	76*			86*	83*	80*
P-SRC Y527		105	102	107			107	103	109			107	105	107
c-MET		100	101	101			102	103	103			100	98	99
P-c-MET		88	92	87*			89	93	85*			92	94	87*
c-KIT		98	98	98			102	102	102			93	100	93
P-c-KIT		91	99	91			98	109	118#			85*	92	79*
PDGFRβ		100	100	91			98	98	98			103	106	103
P-PDGFRβ		89	89	86*			90	90	87*			83*	93	74*
P-JAK2		86*	83*	83*			81*	86*	81*			83*	86*	75*
ERK2		99	99	99			100	101	100			99	100	100
P-ERK1/2		87*	70*	61*¶			84*	85*	77*			87*	82*	76*
JNK1/2		100	101	99			100	100	100			100	104	100
P-JNK1/2		87	89	85*			89	89	82*			86*	91	85*
CD95		100	102	100			106	104	105			99	100	99
FAS-L		102	101	110			101	100	99			107	104	111
HDAC1		97	97	86*			101	101	100			98	99	97
HDAC2		79*	99	68**			73*	98	71*			75*	98	68*
HDAC3		79*	96	69*			77*	96	65**			84*	98	78*
HDAC4		88	93	80*			87	103	81*			85*	101	84*
HDAC5		97	100	94			93	100	93			92	99	87*
HDAC6		80*	105	69**			75*	103	60**			76*	98	74*
HDAC7		87	103	83*			88	100	79*			83*	99	83*
HDAC8		96	99	92			96	100	95			97	101	94
HDAC9		100	98	97			95	100	89			102	100	100
HDAC10		96	99	89			97	97	100			100	98	97
HDAC11		97	96	93			102	98	95			93	100	89
PD-L1		84*	99	74*			82*	99	74*			86*	97	80*
PD-L2		98	97	96			98	98	100			95	97	91
MHCA		114#	96	118#			120#	99	119#			112	103	116#
ODC		90	100	89			90	101	90			95	104	93
IDO1		89	99	85*			91	100	86*			92	100	87*
β-catenin		102	98	95			102	99	96			100	101	100
P-β-catenin		112	111	114*			111	109	114*			103	102	103
YAP		99	99	100			98	99	100			99	98	97
P-YAP S127		112	113#	116#			124#	124#	126#			116#	102	117#
TAZ		104	100	100			102	102	100			100	101	98
P-TAZ S89		112	114#	115#			119#	122#	124#			113#	101	116#

Supplemental Table 2. The impact of GZ17-6.02 and osimertinib on cell signaling in NSCLC cells (part 2). Cells were treated with vehicle control, GZ17-6.02 (2 μM final curcumin), osimertinib (100 nM) or the drugs combined for 6h. Cells were fixed in place and immunostaining performed to determine protein expression and phosphorylation (n = 3 +/-SD) * $p < 0.05$ less than vehicle; ** $p < 0.05$ less than GZ17-6.02 alone; # $p < 0.05$ greater than vehicle.

Vehicle 100%	1975 WT	602	PEM	6+P		1975 AR	602	PEM	6+P		1975 OR	602	PEM	6+P
P-ATM S1980		113#	108	117#			113#	108	116#			114#	109	118#
P-AMPKα T172		112	105	114#			111	107	117#			117#	112	118#
P-mTOR S2448		86*	96	85*			84*	95	82*			88	94	81*
P-mTOR S2481		78*	91	75*			81*	83*	78*			79*	92	75*
P-ULK1 S757		85*	91	82*			89	95	82*			89	96	82*
P-ULK1 S317		108	105	114#			110	105	115#			112	103	120#
P-PERK T980		112	103	116#			114*	102	118#			118#	101	122#
P-eIF2α S51		112	103	118#			111	103	115#			110	101	117#
P-AKT T308		84*	87*	79*			87*	88	84*			83*	87*	79*
P-STAT3 Y705		86*	88	86*			92	95	82*			82*	88	80*
P-STAT5 Y694		89	91	85*			93	94	85*			82*	87*	80*
Beclin1		115#	112	117#			112	111	118#			115#	111	120#
ATG5		115#	109	117#			112	107	117#			115#	110	119#
P-ATG13 S318		112	104	117#			114#	105	118#			115#	106	120#
GRP78		124#	112	130#			120#	109	126#			127#	115#	132#
CHOP		115#	107	117#			112	104	116#			114#	107	119#
PP1		111	109	113#			108	104	110			109	105	111
ERBB1		100	101	98			100	101	100			100	99	100
P-B1		85*	88	75*			87*	93	78*			94	88	84*
ERBB2		100	101	101			100	100	100			99	100	100
P-B2		88	96	75*			86*	97	73*			88	92	85*
ERBB3		99	99	99			99	99	100			100	101	100
P-B3		89	95	85*			91	97	80*			90	93	86*
ERBB4		100	100	100			100	100	101			100	100	100
P-B4		100	100	100			86*	96	83*			86*	95	76*
p70 S6K		100	101	102			100	100	100			100	101	100
p70 S6K T389		91	99	81*			89	97	76*			89	98	78*
P-NFκB S536		87*	85*	83*			88	98	87*			83*	92	80*
P-SRC Y416		95	94	94			86*	92	85*			92	90	86*
P-SRC Y527		105	109	108			107	109	113#			107	112	113#
c-MET		100	100	100			103	100	102			99	100	101
P-c-MET		94	94	90			93	102	93			84*	88	82*
c-KIT		99	99	100			100	101	100			100	101	102
P-c-KIT		85*	94	83*			89	88	82*			89	90	81*
PDGFRβ		101	100	100			98	100	100			99	100	100
P-PDGFRβ		85*	90	82*			82*	84*	80*			81*	98	79*
JAK2		101	102	101			99	100	100			98	98	99
P-JAK2		94	91	89			93	87*	87*			95	89	89
ERK2		100	99	100			98	99	99			100	100	101
P-ERK1/2		86*	95	85*			78*	98	78*			83*	96	80*
CD95		97	100	100			101	101	100			101	100	100
FAS-L		104	108	113#			103	108	113#			105	110	116#

Supplemental Table 3. The impact of GZ17-6.02 and pemetrexed on cell signaling in NSCLC cells. Cells were treated with vehicle control, GZ17-6.02 (2 μM final curcumin), pemetrexed (500 nM) or the drugs combined for 6h. Cells were fixed in place and immunostaining performed to determine protein expression and phosphorylation (n = 3 +/-SD) * $p < 0.05$ less than vehicle; # $p < 0.05$ greater than vehicle.

	VEH	602	PEM	6+P
**H1975 WT**				
**FLIP-s**	**100**	**97**	**101**	**93**
**BCL-XL**	**100**	**90**	**87***	**81***
**MCL-1**	**100**	**85***	**81***	**80***
**ERK2**	**100**	**100**	**100**	**100**
**H1975 OR**				
**FLIP-s**	**100**	**99**	**98**	**98**
**BCL-XL**	**100**	**93**	**90**	**85***
**MCL-1**	**100**	**89***	**84***	**82***
**ERK2**	**100**	**101**	**101**	**101**
**H1650 WT**				
**FLIP-s**	**100**	**91**	**92**	**88**
**BCL-XL**	**100**	**87***	**84***	**83***
**MCL-1**	**100**	**86***	**83***	**80***
**ERK2**	**100**	**100**	**100**	**101**
**H1650 OR**				
**FLIP-s**	**100**	**97**	**93**	**91**
**BCL-XL**	**100**	**87***	**85***	**83***
**MCL-1**	**100**	**90**	**86***	**83***
**ERK2**	**100**	**101**	**100**	**100**

Supplemental Table 4. The impact of GZ17-6.02 and pemetrexed on the expression of cytoprotective proteins in NSCLC cells. Cells were treated with vehicle control, GZ17-6.02 (2 μM final curcumin), pemetrexed (500 nM) or the drugs combined for 6h. Cells were fixed in place and immunostaining performed to determine protein expression (n = 3 +/-SD) * $p < 0.05$ less than vehicle.

1975WT	602	PEM	6+P	1975OR	602	PEM	6+P
1	94	99	86*		96	97	97
2	92	93	86*		90	93	84*
3	91	100	84*		93	98	85*
4	100	100	101		97	100	98
5	99	98	94		94	95	90
6	82*	88	81*		84*	93	83*
7	94	98	94		95	98	97
8	100	101	102		100	101	100
9	101	99	100		96	97	96
10	102	102	98		100	100	102
11	101	101	101		98	101	100
ERK2	100	100	100		102	101	102

Supplemental Table 5. The impact of GZ17-6.02 and pemetrexed on the expression of histone deacetylase (HDAC) proteins in NSCLC cells. Cells were treated with vehicle control, GZ17-6.02 (2 μM final curcumin), pemetrexed (500 nM) or the drugs combined for 6h. Cells were fixed in place and immunostaining performed to determine protein expression (n = 3 +/-SD) * $p < 0.05$ less than vehicle.

	A549			H460			H1437			LLC		
	602	PEM	6+P	602	PEM	6+P	602	PEM	6+P	602	PEM	6+P
PD-L1	64*	85*	65*	66*	87*	69*	78*	85*	72*	68*	90	65*
PD-L2	89	102	99	97	104	93	97	102	97	95	102	94
MHCA	126#	111#	126#	125#	112	128#	125#	112	129#	123#	103	127#
IDO1	97	98	95	85*	95	86*	94	95	92	87	97	89
ODC	90	98	87*	105	103	91	93	96	85*	95	92	91
ERK2	100	99	99	102	100	102	99	99	99	100	98	99

	HCC827ER			H661			H1573			H1299		
	602	PEM	6+P	602	PEM	6+P	602	PEM	6+P	602	PEM	6+P
PD-L1	82*	84*	78*	69*	80*	66*	79*	89	75*	75*	84*	72*
PD-L2	100	100	102	98	102	99	98	104	98	98	100	98
MHCA	130#	123#	145##	124#	113#	132#	125#	111	125#	124#	109	123#
IDO1	69*	78*	70*	75*	83*	74*	82*	92	84*	87*	95	86*
ODC	86*	76*	75*	85*	84*	82*	98	87*	75**	100	99	93
ERK2	99	100	100	101	100	100	101	102	101	100	100	100

	1975WT			1975AR			1975OR			1650WT			1650OR		
	602	PEM	6+P	602	PEM	6+P	602	PEM	6+P	602	PEM	6+P	602	PEM	6+P
PD-L1	72*	86*	71*	84*	89	73**	90	96	82*	74*	85*	71*	86*	94	84*
PD-L2	99	102	100	100	101	96	100	99	97	98	101	98	101	99	98
MHCA	127#	110	125#	128#	108	136#	115#	112	122#	124#	109	123#	116#	107	118#
IDO1	84*	86*	82*	80*	91	78*	91	93	83*	87*	88	86*	90	100	86*
ODC	89	89	80*	81*	80*	84*	89	93	82*	100	91	93	88	89	85*
ERK2	100	100	101	100	100	99	101	100	100	100	99	100	99	101	100

Supplemental Table 6. The impact of GZ17-6.02 and pemetrexed on the expression of immuno-regulatory proteins in NSCLC cells. Cells were treated with vehicle control, GZ17-6.02 (2 μM final curcumin), pemetrexed (500 nM) or the drugs combined for 6h. Cells were fixed in place and immunostaining performed to determine protein expression (n = 3 +/-SD) * $p < 0.05$ less than vehicle; # $p < 0.05$ greater than vehicle.

Figure Legends

Supplemental Figure 1. Representative control data showing siRNA protein expression knock down or protein over-expression. Cells were transfected with plasmids to express the indicated proteins or with siRNA molecules to knock down protein expression. Twenty-four h after transfection cells were fixed in place. In cell immunostaining was performed to detect the levels of each protein and in parallel as a loading control, the total expression of invariant ERK2 (n = 3 +/-SD).

Supplemental Figure 2. Resistance to ERBB1 inhibitors is associated with a reduced ability to form autophagosomes. A. H1975 (wild type sensitive and afatinib-resistant (AR)) were transfected to express LC3-GFP-RFP and subsequently treated with vehicle, osimertinib (100 nM), GZ17-6.02 (2 μM curcumin final) or the drugs in combination for 4h and 8h. The number of intense staining GFP+ and RFP+ punctae were determined randomly in at least 50 cells and the mean number of punctae per cell determined (n = 3 +/-SD). # $p < 0.05$ greater than GZ17-6.02 value; ¶ $p < 0.05$ greater than corresponding value after 4h; ~ $p < 0.05$ less than corresponding value in wild type sensitive cells. **B.** Erlotinib-resistant HCC827 cells were transfected with siRNA molecules to knock down protein levels or with plasmids to express activated forms of mTOR or STAT3 and then subsequently treated with vehicle or [osimertinib (100 nM) + GZ17-6.02 (2 μM curcumin final)] in combination for 4h and 8h. The number of intense staining GFP+ and RFP+ punctae were determined randomly in at least 50 cells and the mean number of punctae per cell determined (n = 3 +/-SD). ¶ $p < 0.05$ greater than corresponding value after 4h; * $p < 0.05$ less than corresponding values in siSCR/CMV transfected cells. **C.** Afatinib-resistant H1975 cells were transfected with siRNA molecules to knock down protein levels or with plasmids to express activated forms of mTOR or STAT3 and then subsequently treated with vehicle or [osimertinib (100 nM) + GZ17-6.02 (2 μM curcumin final)] in combination for 4h and 8h. The number of intense staining GFP+ and RFP+ punctae were determined randomly in at least 50 cells and the mean number of punctae per cell determined (n = 3 +/-SD). ¶ $p < 0.05$ greater than corresponding value after 4h; * $p < 0.05$ less than corresponding values in siSCR/CMV transfected cells.

Supplemental Figure 3. The killing of afatinib-resistant NSCLC cells requires [BAX + BAK] and autophagosome formation and is significantly reduced by expression of activated AKT, activated mTOR or activated MEK1. Afatinib-resistant H1975 cells were transfected with siRNA molecules to knock down protein expression or with plasmids to express regulatory proteins. Subsequently, cells were treated with vehicle or [osimertinib (100 nM) + GZ17-6.02 (2 μM curcumin final)] in combination for 24h. Cell viability was determined by trypan blue exclusion (n = 3 +/-SD). * $p < 0.05$ less than corresponding siSCR/CMV value; ¶ $p < 0.05$ less than corresponding values in all other conditions; § $p < 0.05$ greater than corresponding values in all other manipulated conditions.

Supplemental Figure 4. GZ17-6.02 weakly alters GSH levels and the GSH:GSSG ratio in NSCLC cells. Cells were treated with vehicle control or with GZ17-6.02 (2 μM or 4 μM curcumin final concentration). Cells were isolated 3h-48h afterwards and the total levels of GSH expressed as a percentage of vehicle control at each time point and the ratio of GSH to GSSG determined using a kit purchased from Promega. (n = 3 +/-SD) * $p < 0.05$ less than vehicle control value.

Supplemental Figure 5. In erlotinib-resistant HCC827 cells signaling by ATM enhances autophagosome formation whereas signaling from mTOR suppresses this event. Erlotinib-resistant HCC827 were transfected with siRNA molecules to knock down protein levels or with plasmids to express activated forms of mTOR or STAT3 and then subsequently treated with vehicle or [pemetrexed (500 nM) + GZ17-6.02 (2 μM curcumin final)] in combination for 4h and 8h. The number of intense staining GFP+ and RFP+ punctae were determined randomly in at least 50 cells and the mean number of punctae per cell determined (n = 3 +/-SD). ¶ $p < 0.05$ greater than corresponding value after 4h; * $p < 0.05$ less than corresponding values in siSCR/CMV transfected cells; ∞ $p < 0.05$ less than values in sieIF2α, siAMPKα and caSTAT3.

Supplemental Figure 1

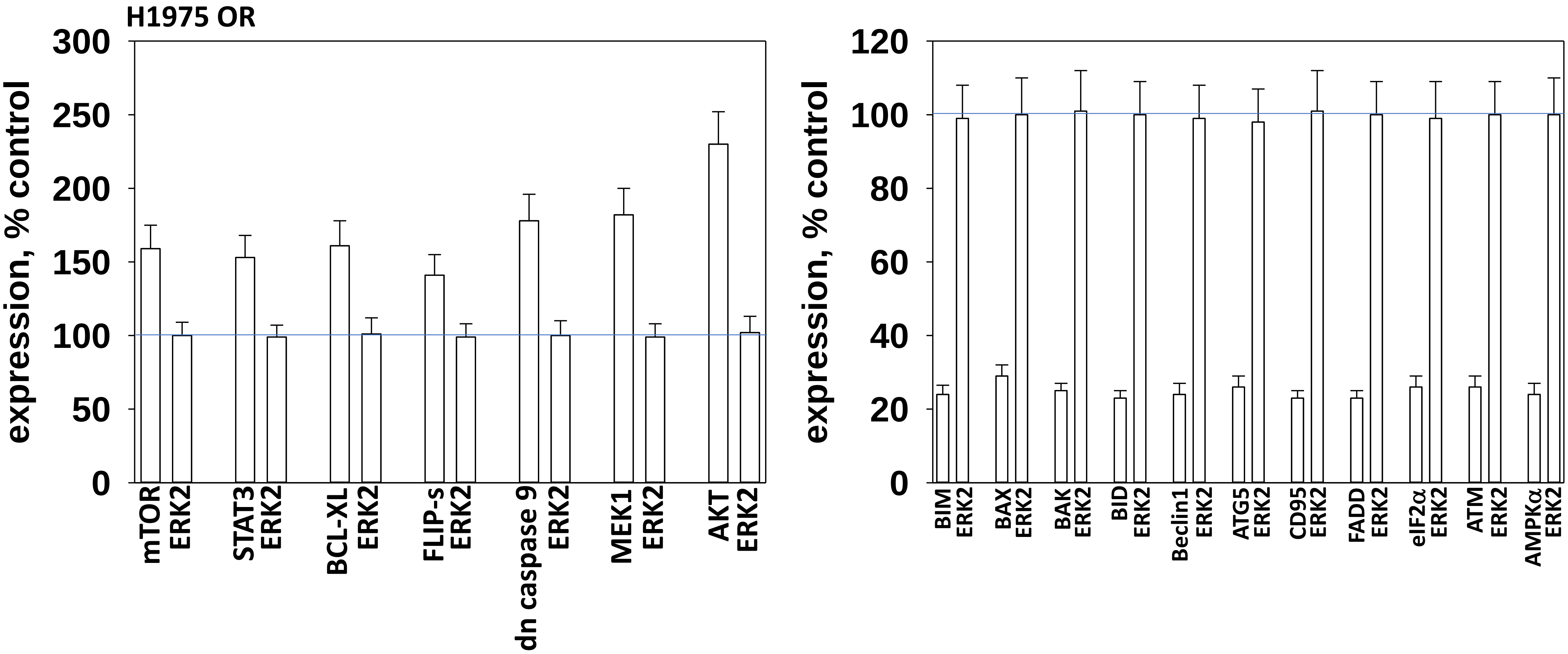

Supplemental Figure 2

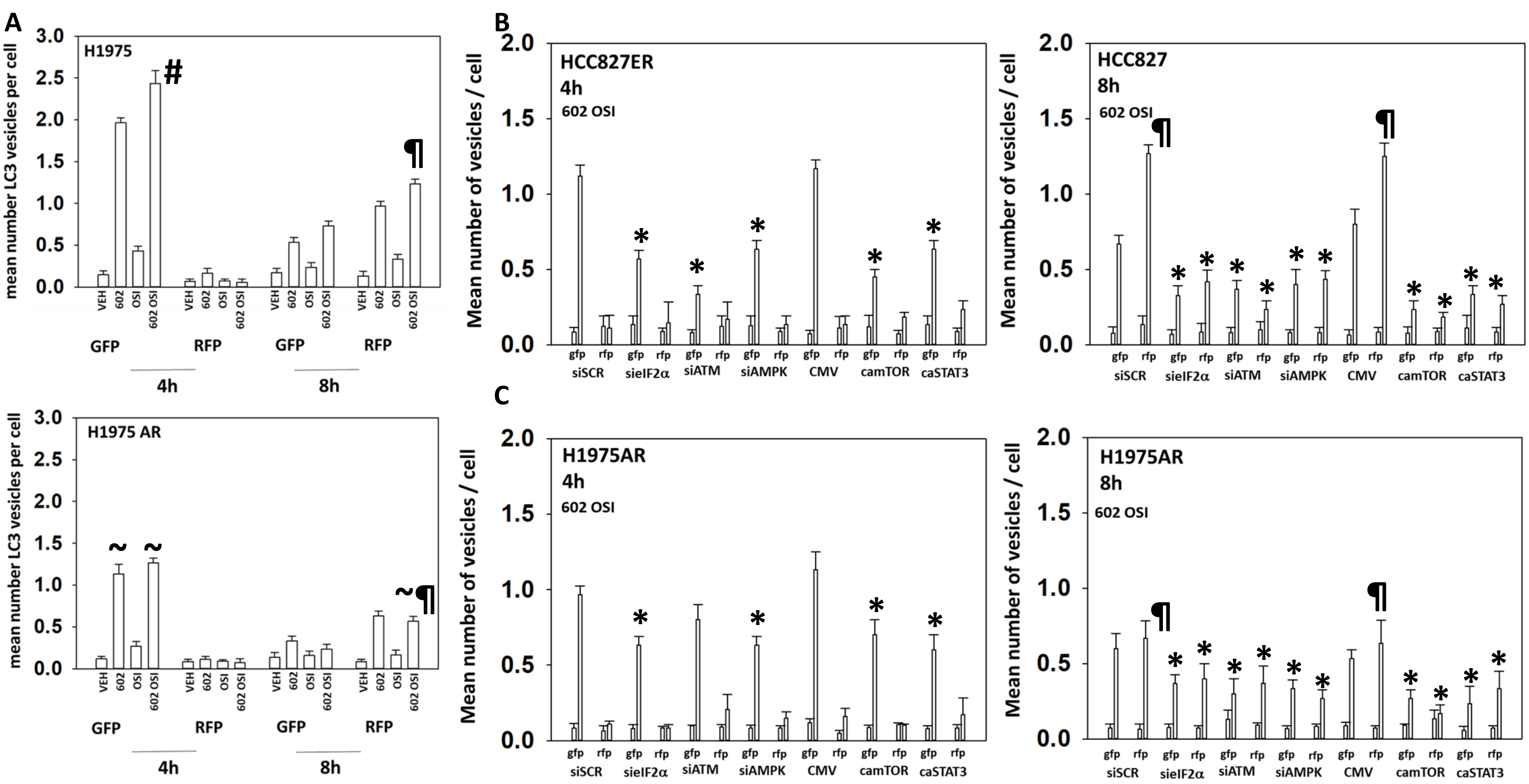

Supplemental Figure 3

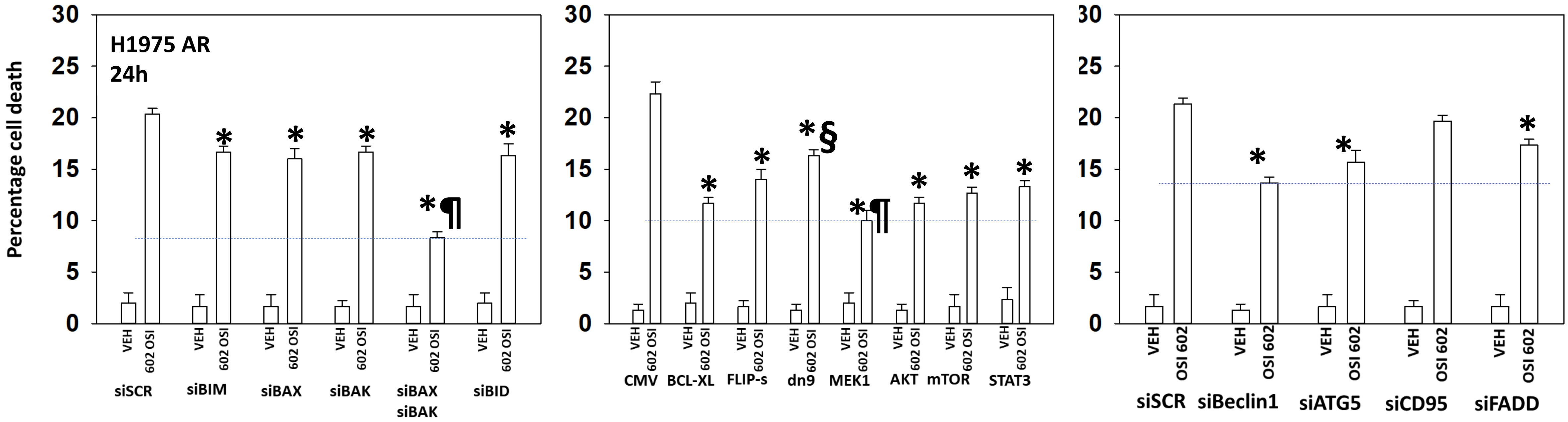

Supplemental Figure 4

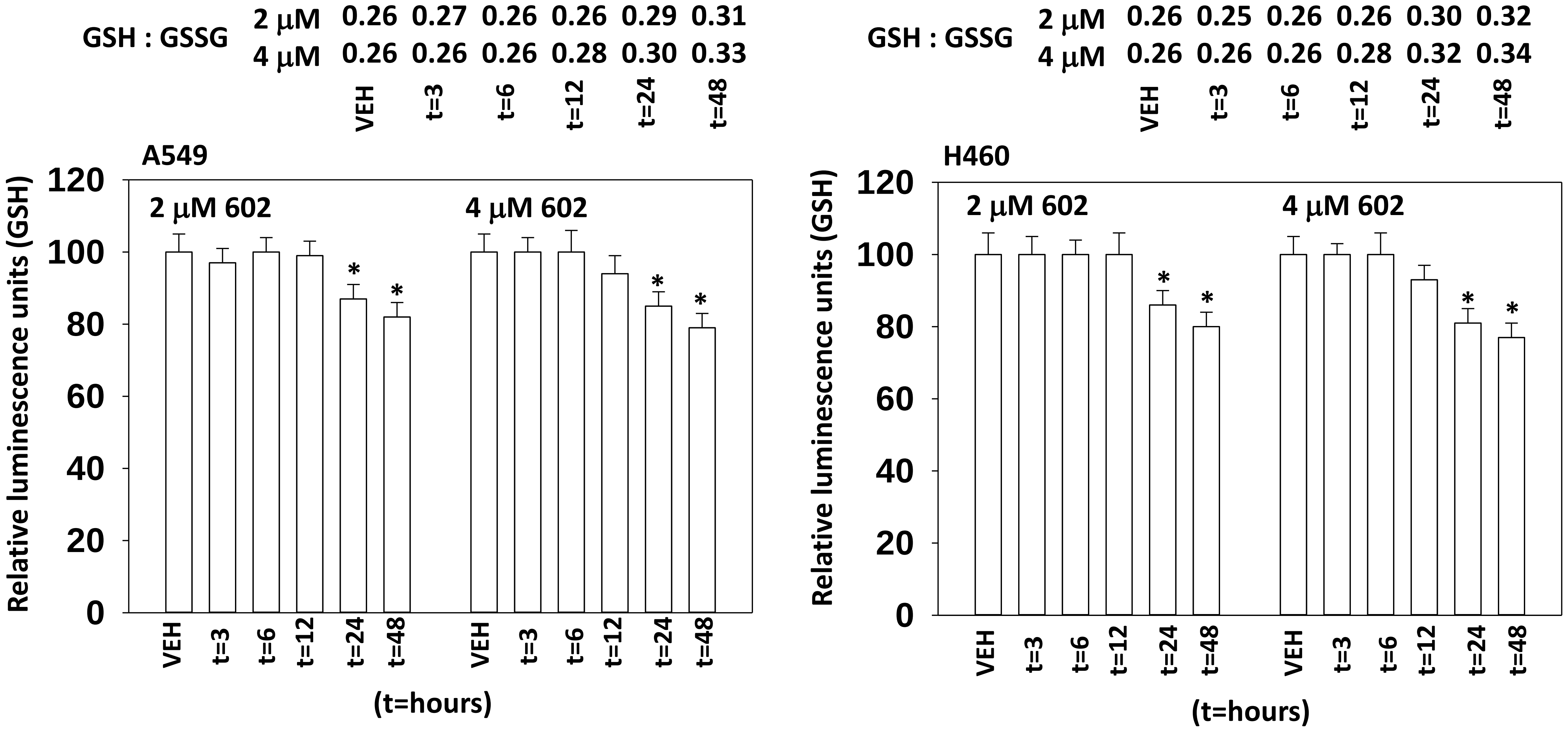

Supplemental Figure 5

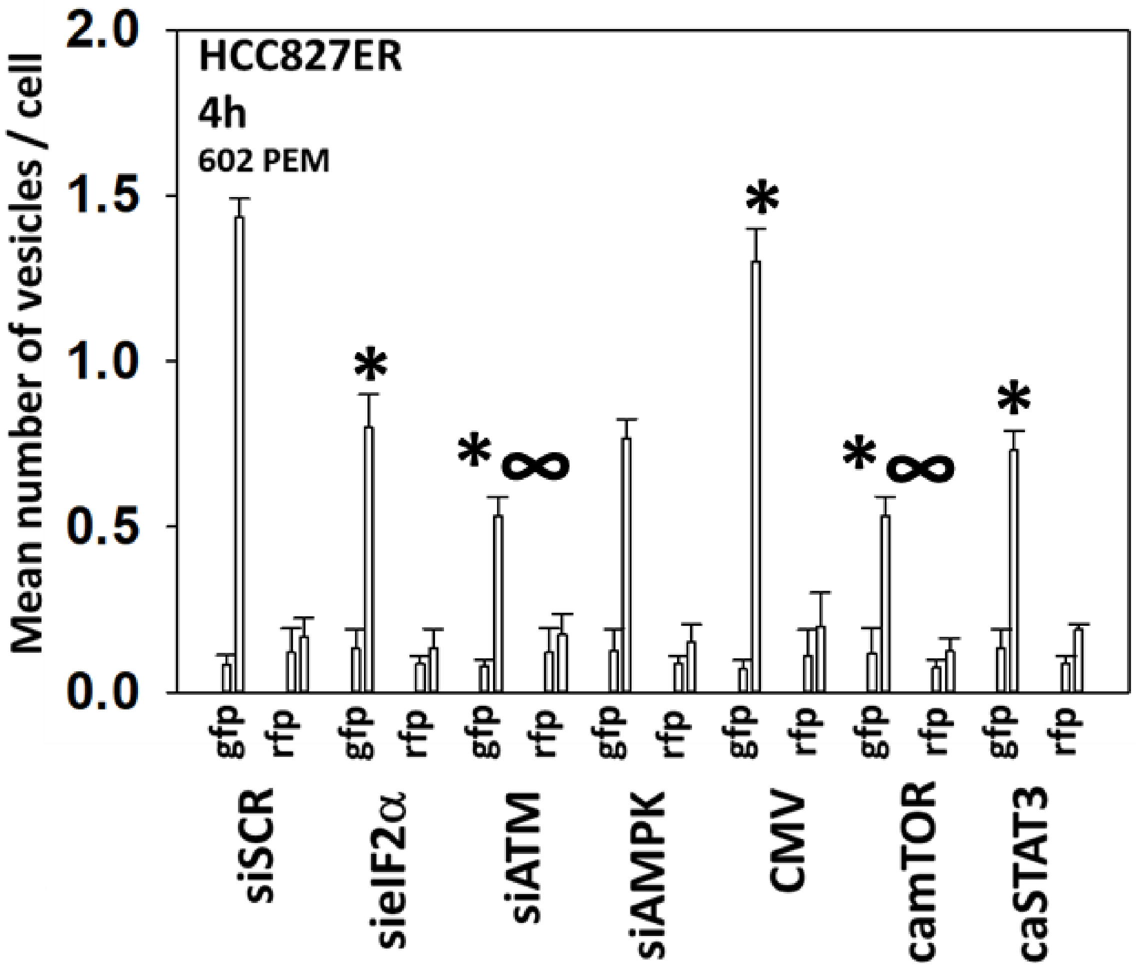

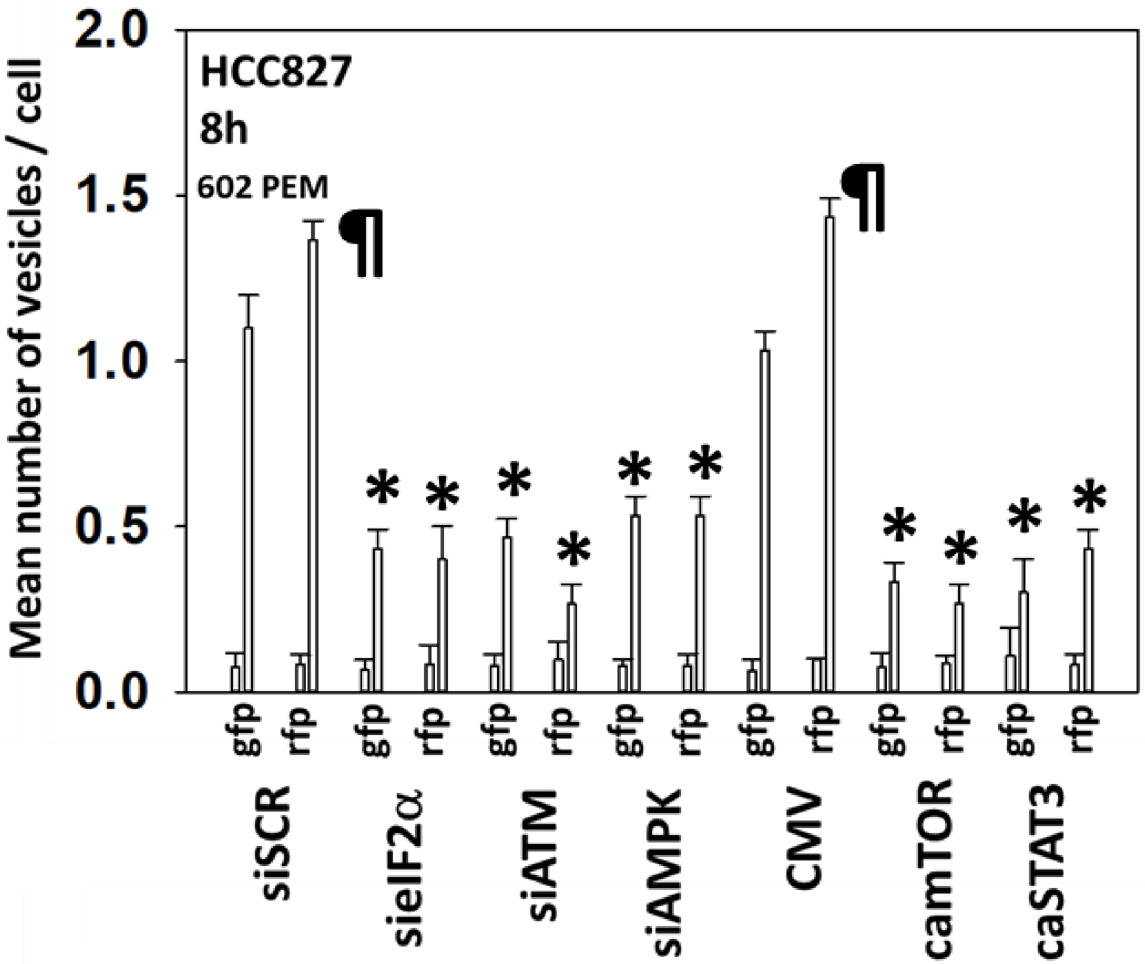

Supplement: Supplementary file 1 [file DataSheet_1.pdf]
